# Comparison of porcine and human vascular diameters for the optimization of interventional stroke training and research

**DOI:** 10.1371/journal.pone.0268005

**Published:** 2022-05-03

**Authors:** Nathalie Mathern, Ehsan Yousefian, Hani Ridwan, Omid Nikoubashman, Martin Wiesmann

**Affiliations:** Department of Diagnostic and Interventional Neuroradiology, University Hospital RWTH Aachen, Aachen, Germany; University Hospital Basel, SWITZERLAND

## Abstract

The branches of the porcine subclavian artery are frequently used in endovascular stroke training and research. This study aimed to determine a porcine weight group, in which the arterial diameters most closely match human cerebral artery diameters, and thus optimize the porcine in-vivo model for neuroendovascular purposes. A group of 42 German Landrace swine (45–74 kg) was divided into four subgroups according to their weight. Angiographic images of the swine were used to determine the arterial diameter of the main branches of the subclavian artery: axillary artery, brachial artery, external thoracic artery, subscapular artery (at two different segments), suprascapular artery, caudal circumflex humeral artery, thoracodorsal artery, and circumflex scapular artery. The porcine arterial diameters were correlated with animal weight and compared to luminal diameters of human arteries which are commonly involved in stroke: internal carotid artery, basilar artery, vertebral artery, middle cerebral artery and M2 branches of the middle cerebral artery. Swine weight was positively correlated with porcine arterial diameter. The most conformity with human arterial diameters was found within the two heavier porcine groups (55–74 kg). We suggest the use of swine with a weight between 55–59.7 kg, as lighter animals show less similarity with human arterial diameters and heavier animals could cause more problems with manipulation and handling.

## Introduction

The domestic swine is a widely used in-vivo model in endovascular research. In neuroradiology, it is used for interventional training courses, the testing of new devices, and for the development of new therapies [1–7]. Porcine animal models have gained increasing popularity due to similar anatomical, hemodynamic, and hemostatic conditions to humans [8]. Their body size allows the insertion of human standard-sized devices [9,10]. The porcine internal carotid artery passes through the rete mirabile, which is an anatomical characteristic of this model [8]. The diameter of the arteries forming this small network make an interventional access beyond it impossible [11]. Instead, branches of the subclavian artery can be used for interventional training and research [2]. Although swine are commonly used in-vivo models, published data on the peripheral porcine vasculature is limited [3]. Moreover, although it is common to use swine before they are fully grown and their corpus length is too long for human devices, there are no data on the ideal weight of swine for endovascular stroke models.

It is safe to assume that arterial diameters increase with weight in animals which are not yet fully grown. In the present study, we therefore determined the diameters of the main branches of the subclavian artery in 42 not yet fully grown German Landrace swine of various weight. We compared the diameter of the porcine vessels to the diameter of the human basilar, vertebral, internal carotid, and M1 and M2 segments of the middle cerebral artery, which are commonly involved in human stroke [12]. The purpose of this study was to find an optimal weight range of German Landrace swine, in which the porcine arterial diameter is closest to the diameter of the selected human arteries, with the aim to optimize the usefulness of the porcine in-vivo model for endovascular research.

### Animals

There were 42 German Landrace swine (Gerd Heinrichs, Heinsberg-Karken, Germany) included into this retrospective study. Measurements were performed on female swine with weights from 45 kg to 74 kg and average ages between 3 and 5 months.

All animals included in this study were part of different neuroradiological short-term experiments, with a maximum duration of 10 hours. The experiments were performed in accordance with the (blinded) German legislation governing animal studies following the “Guide for the Care and Use of Laboratory Animals” (National Research Council, 8^th^ edition, 2011) and the “Directive 2010/63/EU on the Protection of Animals Used for Scientific Purposes” (EU Official Journal, 2010). Official permission was granted from the (blinded) governmental animal care and use office (Landesamt für Natur, Umwelt und Verbraucherschutz Nordrhein-Westfalen, Recklinghausen, Germany, approval number: 81.-02.05.40.18.078). Institutional guidelines for animal welfare and experimental implementation were followed. Animals were housed under controlled environmental conditions (20°C ±1°C, 12:12h light/dark cycle). The acclimatization period before starting the experiments was 1–2 weeks. Besides from fasting shortly before the experiments, all swine received feed and water ad libitum. The swine were housed in groups of 2–4 animals. Litter and materials for examination and manipulation were available.

Standard anesthesia treatment provided premedication with azaperone (Stresnil 40 mg ad. us. vet.; Sanochemia Pharmazeutika AG, Neufeld, Austria), atropin (Atropinsulfat; B. Braun Melsungen AG, Melsungen, Germany), and ketamine (10% Ketavet ad us. vet.; Zoetis Deutschland GmbH, Berlin, Germany). The swine were intubated and connected to mechanical ventilation with an oxygen / air mixture. Anesthesia was maintained either with propofol (Propofol 2% MCT Fresenius; Fresenius Kabi Deutschland GmbH, Bad Homburg, Germany) or with isoflurane, dependent on the operation room equipment. Fentanyl (Fentanyl-Janssen 0.5 mg; Janssen-Cilag GmbH, Neuss, Germany) was continuously administered for analgesia. During the experiments, vital parameters of the animals were monitored. The swine received an intravenous dose of ASA (Aspirin i.v. 500 mg; Bayer Vital, Leverkusen, Germany) and heparin (Heparin- Natrium 5000 I.E 10.2 ml; Ratiopharm, Ulm, Germany). The animals were fixed in supine position, with the forelimbs extended and fixed next to the head.

An 8-French femoral artery sheath was placed percutaneously after induction of anesthesia. The sheath was flushed with heparinized saline. 5-French to 8-French catheters and guidewires, which are in regular use in human angiography, were used to make access to the subclavian artery. Digital subtraction angiography of the forelimb was performed by using iopamidol (Solutrast 300, 300 mg/ml; Bracco Imaging Deutschland GmbH, Konstanz, Germany) as a contrast agent. At the end of the experiments, or at a maximum duration of 10 hours, animals were euthanized with an intravenous injection of natrium-pentobarbital (Narcoren 16g/100 ml; Merial GmbH, Hallbergmoos, Germany).

## Material and methods

### Measurements and imaging

The swine were assigned to different acute studies for interventional training or device testing. All images were reviewed retrospectively.

The experiments were performed in accordance with the (blinded) German legislation governing animal studies following the “Guide for the Care and Use of Laboratory Animals” (National Research Council, 8th edition, 2011) and the “Directive 2010/63/EU on the Protection of Animals Used for Scientific Purposes” (EU Official Journal, 2010). Official permission was granted from the (blinded) governmental animal care and use office and written consent was given (Landesamt für Natur, Umwelt und Verbraucherschutz Nordrhein-Westfalen, Recklinghausen, Germany, approval number: 81.-02.05.40.18.078).

**Fig 1:**
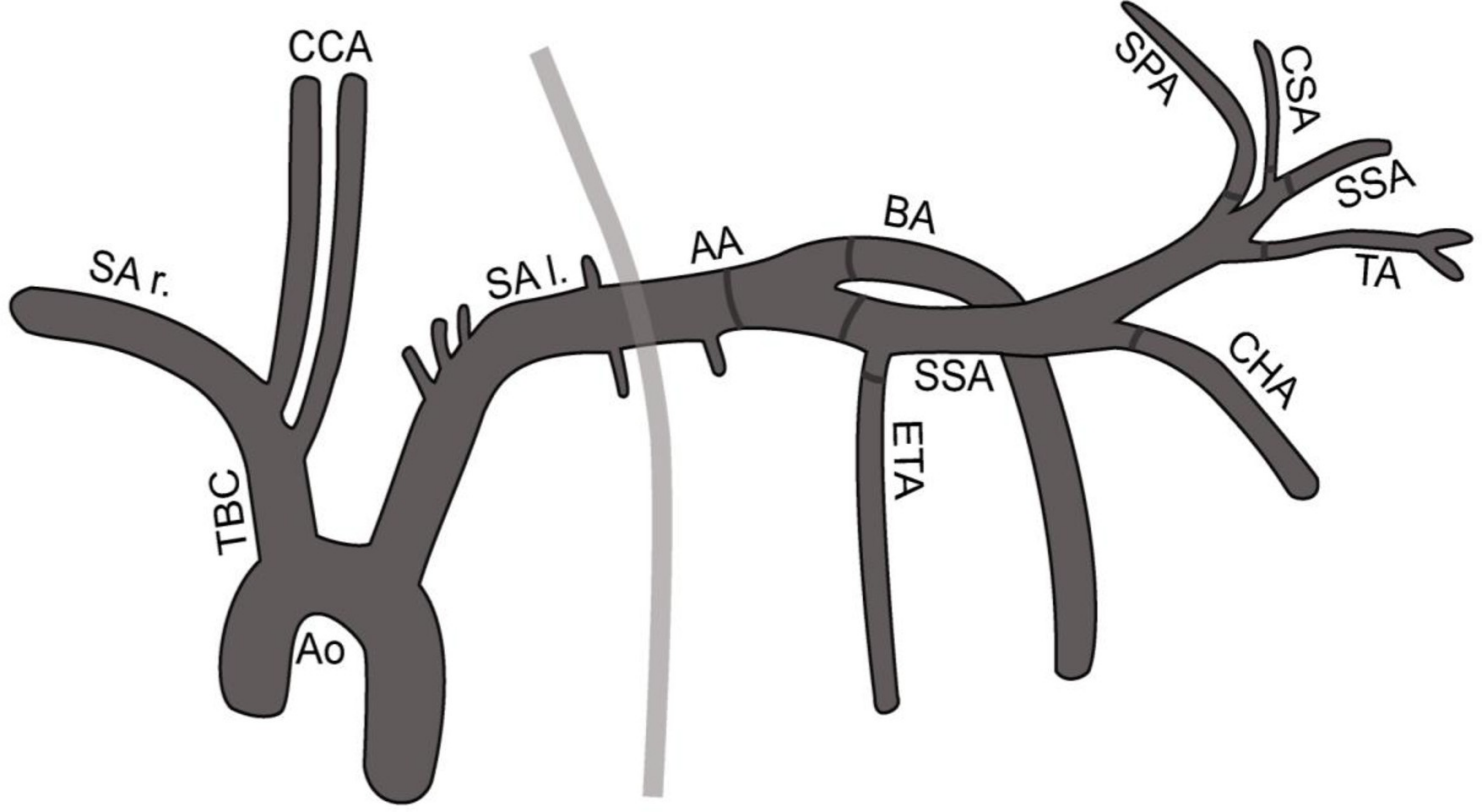
Simplified figure of the aortic arch and the main branches of the porcine subclavian artery. The black lines represent the areas in which the arterial diameters were determined. The light grey line represents the area in which the subclavian artery leaves the chest. Ao: Aorta; CCA: Common Carotid Artery; TBC: Brachiocephalic trunk; SA l./ SA r.: Subclavian Artery left/ right; AA: Axillary Artery; BA: Brachial Artery; ETA: External Thoracic Artery; SSA: Subscapular Artery; SPA: Suprascapular Artery; CSA: Circumflex Scapular Artery; TA: Thoracodorsal Artery; CHA: Caudal Circumflex Humeral Artery.

**Fig 2:**
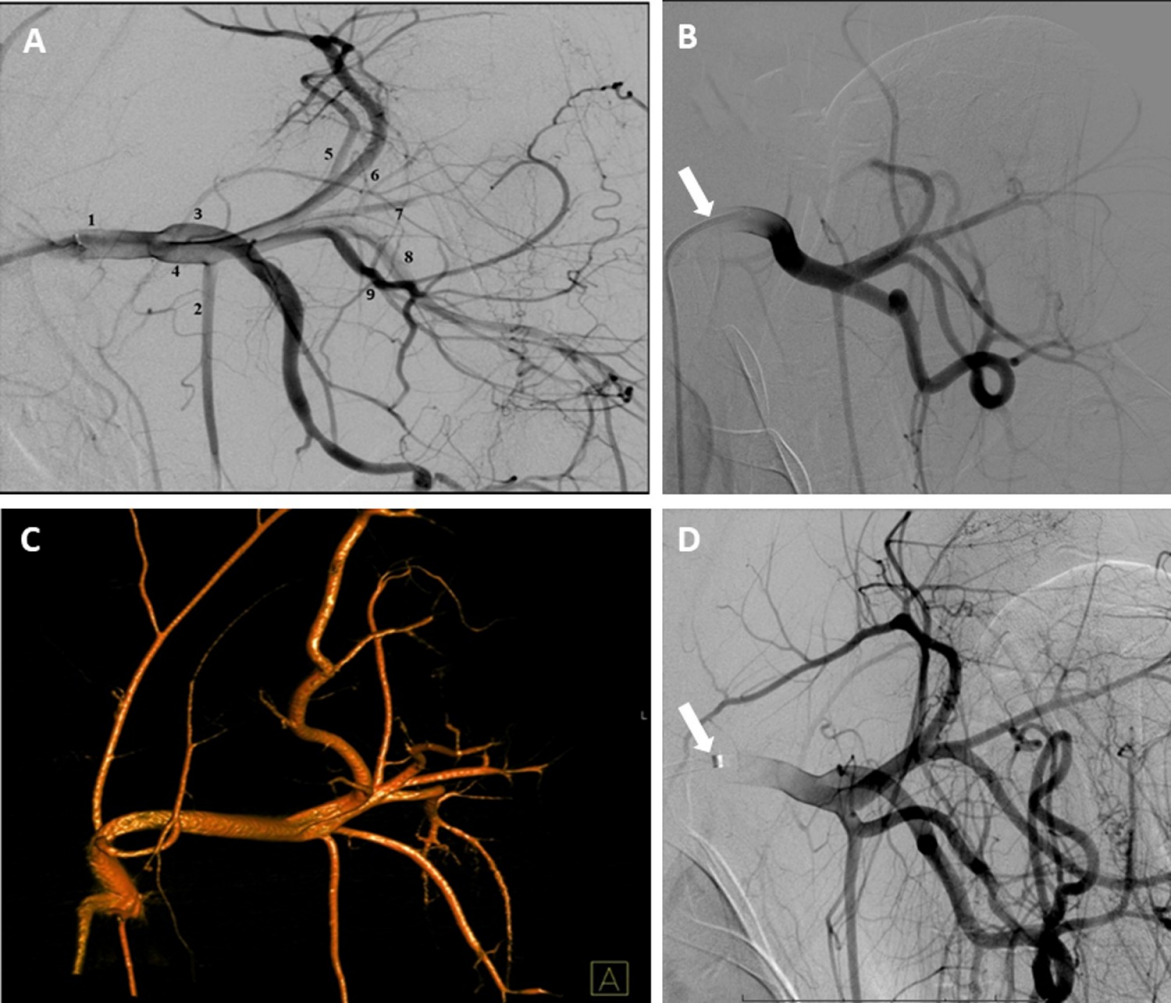
Images of digital subtraction angiography via endovascular catheters (arrows) placed in the left axillar artery (A, B, D). Three-dimensional angiographic reconstruction of the subclavian artery and the main branches of the porcine shoulder (C). 1. Axillary Artery, 2. External Thoracic Artery, 3. Brachial Artery, 4. Subscapular Artery (first segment), 5. Circumflex Scapular Artery, 6. Suprascapular Artery, 7. Subscapular Artery (second segment), 8. Thoracodorsal Artery, 9. Caudal Circumflex Humeral Artery.

Images were acquired using either a Siemens (Arcadis Avantic/ Arcadis ORBIC, Siemens Healthcare GmbH, Erlangen, Germany) or a Ziehm (Ziehm Vision, Ziehm Imaging GmbH, Nürnberg, Germany) C-arm angiography unit. A vascular analysis program (IntelliSpace PACS 4.4 Enterprise, Philips GmbH, Eindhoven, The Netherlands) was used to determine the diameters of the arterial forelimb vasculature. A careful calibration process of the angiographic images, based on the size of the guide catheter introduced into the subclavian artery, was performed to increase the accuracy of the measurements. Diameters of the following arteries were determined: axillary artery, brachial artery, external thoracic artery, subscapular artery (first segment: after originating from the axillary artery; second segment: after branching of the circumflex scapular artery), suprascapular artery, circumflex scapular artery, thoracodorsal artery, and caudal circumflex humeral artery (Figs 1 and 2). The data of the human arterial diameters were based on own measurements in 100 stroke patients treated in our clinic (unpublished data). After a accurate calibration process, the diameters were determined out of digital subtraction angiography images.

### Statistical analysis

Out of a total number of 65 swine, 42 were included into this retrospective study. The data of the excluded swine were not considered to be measurable accurately. Four percentiles were formed, dividing the group of 42 swine into four subgroups according to their weight (Group A: 45–51.8 kg, n = 10; Group B: 51.8–55 kg, n = 10; Group C: 55–59.7 kg, n = 12; Group D: 59.7–74 kg, n = 10). Data were analyzed with SPSS statistics 25 software (IBM, San Jose, California, USA) to compare the groups according to vessel diameter and weight. The mean and standard deviation were calculated for each parameter. The Pearson correlation coefficient (r) was used to evaluate the correlation between porcine weight and arterial diameter. A positive value meant a positive correlation between both variables, a negative value indicated a negative correlation. The strength of the linear association between swine weight and arterial diameter was based on the r value: ± 0.1 to ± 0.3, small correlation; ±0.3 to ±0.5, moderate correlation; and ± 0.5 to ± 1.0, strong correlation. Student’s t-test was used for analyzing statistical differences regarding porcine and human arterial diameters. A p-value of less than 0.05 was considered as significant.

## Results

**Fig 3:**
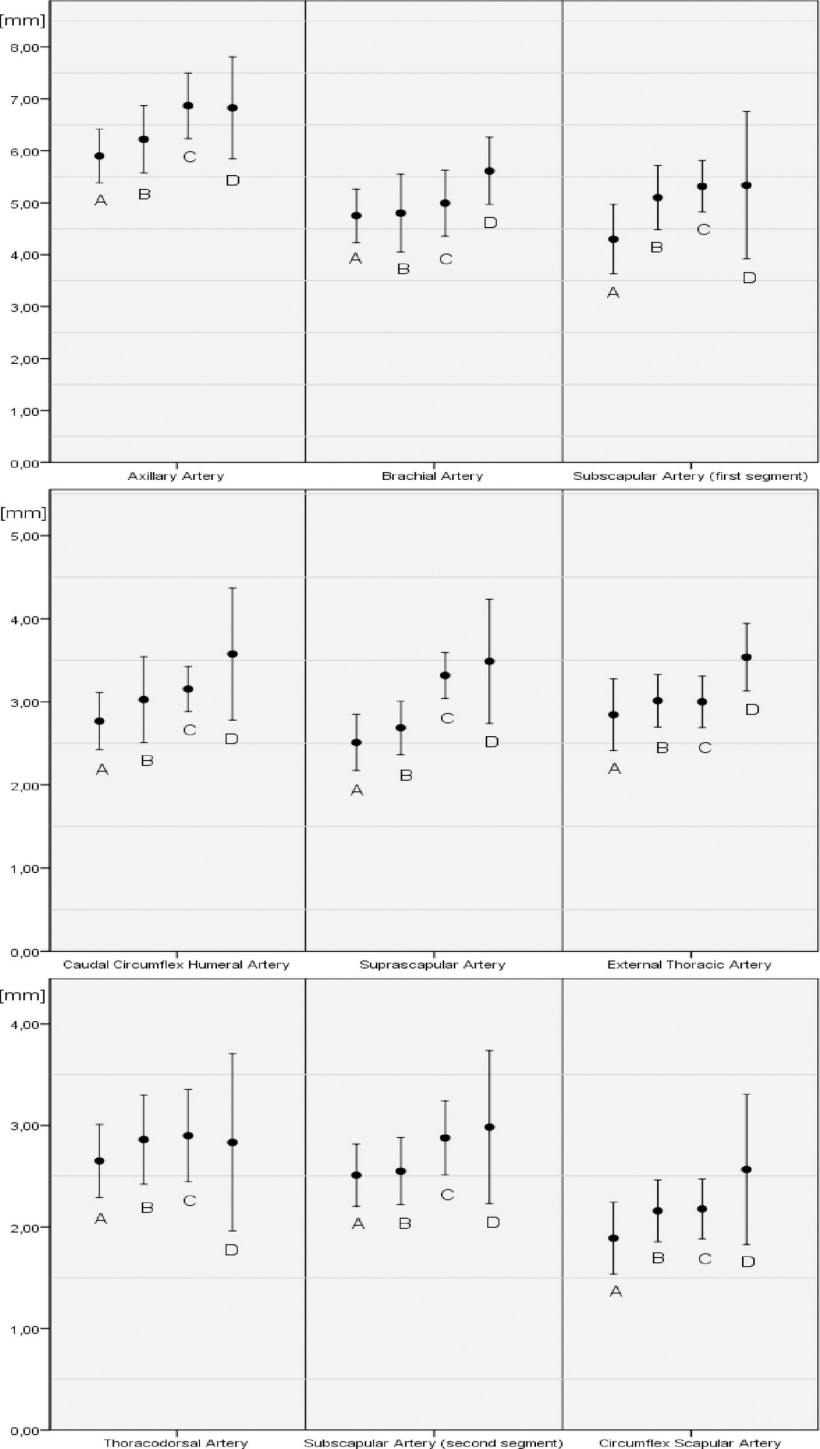
The graphic shows the mean arterial diameter (in mm) and the standard deviation of arteries of the forelimb per porcine weight group. Swine were divided into four subgroups according to their weight (Group A: 45–51.8 kg; Group B: 51.8–55 kg; Group C: 55–59.7 kg; Group D: 59.7–74 kg).

Mean arterial diameters of the measured arteries are presented in Fig 3. Statistical analysis showed, that our hypothesis was confirmed and the arterial diameters of the swine increased with increasing body weight (Table 1). Eight of nine measured arterial diameters showed a positive correlation with the porcine weight. Six of the selected arteries showed a statistically significant, moderate positive correlation with the swine weight: axillary artery (r = .363, p = .023), brachial artery (r = .424, p = .005), external thoracic artery (r = .461, p = .002), circumflex scapular artery (r = .476, p = .003), suprascapular artery (r = .409, p = .007), and caudal circumflex humeral artery (r = .405, p = .014). The subscapular artery (first segment) showed a moderate, but statistically not significant positive correlation with the swine weight (r = .312, p = .053). There was a small positive correlation with swine weight for the second segment of the porcine subclavian artery (r = .260, p = .110). No diameter to weight correlation was found for the thoracodorsal artery (r = .025, p = .876).

**Table 1:** Mean arterial diameters (±1 SD) according to quantitative vascular angiography depending on swine weight.

	Axillary Artery	Brachial Artery	External Thoracic Artery	Subscapular Artery (first segment)	Subscapular Artery (second segment)	Circumflex Scapular Artery	Supra- scapular Artery	Thoraco- dorsal Artery	Caudal Circumflex Humeral Artery
Group A	6.0±0.6	4.7±0.8	2.8±0.5	4.3±0.8	2.5±0.4	1.9±0.5	2.6±0.5	2.7±0.5	2.8±0.5
Group B	6.2±0.8	4.8±0.9	3.0±0.3	5.1±0.8	2.6±0.5	2.2±0.4	2.7±0.4	2.9±0.6	3.0±0.6
Group C	6.9±1.0	5.0±1.0	3.0±0.4	5.3±0.8	3.0±0.5	2.2±0.4	3.3±0.4	3.0±0.6	3.2±0.4
Group D	6.8±1.1	5.7±0.8	3.4±0.6	5.4±1.6	2.8±0.7	2.6±0.6	3.3±0.9	2.7±0.8	3.6±0.9
Pearson correlation (r)	.363	.424	.461	.312	.260	.476	.409	.025	.405
p-value	.023	.005	.002	.053	.110	.003	.007	.876	.014

Swine were divided into four subgroups according to their weight (Group A: 45–51.8 kg; Group B: 51.8–55 kg; Group C: 55–59.7 kg; Group D: 59.7–74 kg). The Pearson correlation coefficient (r) indicates the correlation between swine weight and arterial diameter. Results indicate that mean arterial diameters of six arteries (Axillary artery, brachial artery, external thoracic artery, circumflex scapular artery, suprascapular artery, and caudal circumflex humeral artery) increase with weight respectively age of the swine.

The largest determined vessel of the porcine forelimb was the axillary artery (6.0 mm (group A) - 6.9 mm (group C)). The smallest determined vessel of the porcine forelimb was the circumflex scapular artery (1.9 mm (group A) - 2.6 mm (group D)). For the comparison with human arterial diameters six arteries which are commonly involved in human stroke were used (Table 2). Mean and standard deviation (SD) of arterial diameters were determined as follows: internal carotid artery (ICA): 5.2 ± 0.5 mm; basilar artery (BA): 3.1 mm ± 0.4 mm; vertebral artery (VA): 3.2 ± 0.5 mm; middle cerebral artery (MCA, M1 segment): 2.4 ± 0.2 mm; middle cerebral artery (MCA, M2 segment, first branch): 1.6 ± 0.2 mm; middle cerebral artery (MCA, M2 segment, second branch): 1.4 ± 0.2 mm. Measurements for the vertebral artery were taken at the level of the V3 segment. Each porcine weight group had similarities with four (ICA, VA, BA, MCA (M1)) of the selected human arteries. The smaller human arteries, the branches of the M2 segment of the middle cerebral artery, showed no statistical similarity to the selected porcine vessels, with exception of the porcine circumflex scapular artery, which did not differ significantly from the first branch of the M2 segment (p = .097). Quantitatively, the arteries of group A showed the fewest (n = 7) and the arteries of group D the most (n = 16) statistically significant similarities to the human arteries. The highest conformity with the human ICA was found within the subscapular artery (first segment) of group B (p = .718). The human BA showed the highest conformity with the thoracodorsal artery of group C (p = .781), the human VA with the caudal circumflex humeral artery of the same group (p = .716). The highest similarity with the human MCA was found within the second segment of the subscapular artery of group A (p = .437).

**Table 2:** Agreement of porcine mean arterial diameters (± 1 SD) with mean arterial diameters in human stroke patients.

			Axillary Artery	Brachial Artery	External Thoracic Artery	Subscapular Artery (first segment)	Subscapular Artery (second segment)	Circumflex Scapular Artery	Suprascapular Artery	Thoracodorsal Artery	Caudal Circumflex Humeral Artery
Group A	Mean Diameter (mm)±1 SD		6.0±0.6	4.7±0.8	2.8±0.5	4.3±0.8	2.5±0.4	1.9±0.5	2.6±0.5	2.7±0.5	2.8±0.5
	p-value	ICA	.006	.085	.000	.007	.000	.000	.000	.000	.000
		BA	.000	.000	.125	.001	.002	.000	.007	.020	.057
		VA	.000	.000	.049	.002	.001	.000	.002	.007	.020
		MCA (M1)	.000	.000	.041	.000	.437	.010	.258	.151	.040
		MCA (M2_1)	.000	.000	.000	.000	.000	.097	.000	.000	.000
		MCA (M2_2)	.000	.000	.000	.000	.000	.012	.000	.000	.000
Group B	Mean Diameter (mm)±1 SD		6.2±0.8	4.8±0.9	3.0±0.3	5.1±0.8	2.6±0.5	2.2±0.4	2.7±0.4	2.9±0.6	3.0±0.6
	p-value	ICA	.007	.239	.000	.718	.000	.000	.000	.000	.000
		BA	.000	.000	.368	.000	.004	.000	.014	.248	.742
		VA	.000	.000	.090	.000	.002	.000	.004	.114	.450
		MCA (M1)	.000	.000	.000	.000	.332	.108	.019	.042	.024
		MCA (M2_1)	.000	.000	.000	.000	.000	.002	.000	.000	.000
		MCA (M2_2)	.000	.000	.000	.000	.000	.000	.000	.000	.000
Group C	Mean Diameter (mm)±1 SD		6.9±1.0	5.0±1.0	3.0±0.4	5.3±0.8	3.0±0.5	2.2±0.4	3.3±0.4	3.0±0.6	3.2±0.4
	p-value	ICA	.000	.484	.000	.616	.000	.000	.000	.000	.000
		BA	.000	.000	.445	.000	.486	.000	.066	.781	.663
		VA	.000	.000	.141	.000	.211	.000	.268	.438	.716
		MCA (M1)	.000	.000	.001	.000	.005	.120	.000	.007	.000
		MCA (M2_1)	.000	.000	.000	.000	.000	.002	.000	.000	.000
		MCA (M2_2)	.000	.000	.000	.000	.000	.000	.000	.000	.000
Group D	Mean Diameter (mm)±1 SD		6.8±1.1	5.7±0.8	3.4±0.6	5.4±1.6	2.8±0.7	2.6±0.6	3.3±0.9	2.7±0.8	3.6±0.9
	p-value	ICA	.002	.091	.000	.717	.000	.000	.000	.000	.002
		BA	.000	.000	.136	.003	.348	.051	.458	.227	.200
		VA	.000	.000	.303	.003	.208	.025	.671	.135	.301
		MCA (M1)	.000	.000	.000	.000	.138	.377	.012	.269	.010
		MCA (M2_1)	.000	.000	.000	.000	.002	.002	.000	.004	.001
		MCA (M2_2)	.000	.000	.000	.000	.001	.001	.000	.001	.000

Swine were divided into four subgroups according to their weight (Group A: 45–51.8 kg; Group B: 51.8–55 kg; Group C: 55–59.7 kg; Group D: 59.7–74 kg). Data of human patients are based on own measurements in 100 stroke patients treated in our clinic (unpublished data, see Materials and Methods). The p-value (Student’s t-test) is related to the comparison between porcine arterial diameters and selected human arterial diameters: internal carotid artery (ICA); basilar artery (BA); vertebral artery (VA); M1 branch of the middle cerebral artery (MCA M1); first M2 branch of the middle cerebral artery (MCA M2_1); second M2 branch of the middle cerebral artery (MCA M2_2).

## Discussion

When working with in-vivo models, the selection of a suitable animal model is a major part to achieve the respective aims of research [13]. To best of our knowledge, our study defines for the first time typical measures for the arteries of the porcine forelimb. We found similarities between the diameter of these arteries and diameters of the human brain arteries, which are commonly involved in human stroke. The growing field of neuroendovascular interventions is accompanied by more complex procedures which increases the need for a suitable training model. The use of swine as animal model for hands-on training and research has become popular in the last years. They offer a realistic training setting as they have similar physiological parameters to humans and allow for complication management training [2,8]. Schwartz et al. suggest the use of the peripheral arteries of the domestic cross-bred swine for device testing, because of a similar size and access in comparison to those of human vessels [14]. Published data on porcine arterial diameters are limited, although adequate anatomical knowledge of the arteries and their diameters is highly relevant for the selection of a suitable animal model for each in-vivo study [3]. With the precise knowledge about vessel sizes, more detailed results could be received, whereas a false choice of vessels may lead to incorrect study results and could cause injuries in the vessel wall of the animal [15]. In the field of neuroradiology, detailed knowledge of the porcine vessels allows for targeted placement of thrombi and the use of endovascular devices with the right size. Situations known from human interventions can be recreated and trained, which increases the usefulness of this model by making the training more realistic.

Our results show, that arterial diameters of group D (59.7–74 kg), but also group C (55–59.7 kg), were qualitatively and quantitatively most similar to those of humans. We therefore suggest the use of swine with a greater body weight for the imitation of the larger human brain arteries (ICA, BA, VA, ACM). The arterial diameters of swine with a lower body weight were often undersized (group A) or not offering enough suitable vessels with similar diameter (group B). Smaller cerebral human arteries, such as M2 branches of the ACM, could not be well reproduced with the selected arteries of swine with a body weight between 45 kg to 75 kg. As our results show, there was a positive correlation between porcine body weight and arterial diameter. Thus, the use of a lighter German Landrace swine (<45 kg) could possibly cover the vessel sizes of human M2 branches of the ACM. Another option could be the use of smaller side branches of the main vessels, which were not measured in our study.

When working with large animal models it should not be ignored that the larger and heavier an animal is, its handling and manipulation can be accompanied by more problems [12]. It should also not be disregarded that the fast growing corpus length of the swine could render it too long for human devices at a certain point. However, we used standard-sized human material without any problems in the swine included in this study. For this reason, swine with a body weight between 55 kg to 59.7 kg (group C) may represent the best compromise of all groups. However, variations of human anatomy, or different neurointerventional approaches may need to be considered for the selection of a suitable animal model [16,17].

## Limitations

The proposed porcine vessels have similar diameters to human vessels. However, wall structures and tortuosity are different, which means that it is not possible to recreate maximally realistic scenarios [10,18]. A major limitation of our study was that the used swine breed, German Landrace, has not yet been widely used in neuroendovascular trials. Differences in anatomy and growth ensure that transfer of the data to other porcine breeds is not directly possible.

## Conclusions

The results of this study may serve as reference points for future endovascular training and research. For porcine models of stroke, swine with a bodyweight between 55 kg to 59.7 kg may represent a suitable compromise between anatomical conformity and everyday suitability for laboratory use.

## Supporting information

S1 Dataset (XLSX)
